# Characterization of the Pyrroloquinoline Quinone Producing *Rhodopseudomonas palustris* as a Plant Growth-Promoting Bacterium under Photoautotrophic and Photoheterotrophic Culture Conditions

**DOI:** 10.3390/ijms241814080

**Published:** 2023-09-14

**Authors:** Shou-Chen Lo, Shang-Yieng Tsai, Wei-Hsiang Chang, I-Chen Wu, Nga-Lai Sou, Shih-Hsun Walter Hung, En-Pei Isabel Chiang, Chieh-Chen Huang

**Affiliations:** 1 Department of Life Sciences, National Chung Hsing University, Taichung 402202, Taiwan; scl@dragon.nchu.edu.tw (S.-C.L.); seiken.public@gmail.com (S.-Y.T.); ben0306jamin@gmail.com (W.-H.C.); kyle172224@gmail.com (I.-C.W.); walter030170@gmail.com (S.-H.W.H.); 2 Department of Food Science and Biotechnology, National Chung Hsing University, Taichung 402202, Taiwan; looksusan2013@gmail.com (N.-L.S.); chiangisabel@nchu.edu.tw (E.-P.I.C.); 3 Institute of Plant and Microbial Biology, Academia Sinica, Taipei 115201, Taiwan; 4 Program in Microbial Genomics, National Chung Hsing University, Taichung 402202, Taiwan; 5 Innovation and Development Center of Sustainable Agriculture, National Chung Hsing University, Taichung 402202, Taiwan

**Keywords:** *Rhodopseudomonas palustris*, purple non-sulfide bacterium, pyrroloquinoline quinone, *Arabidopsis*, autotrophic culture, endophyte

## Abstract

*Rhodopseudomonas palustris* is a purple non-sulfide bacterium (PNSB), and some strains have been proven to promote plant growth. However, the mechanism underlying the effect of these PNSBs remains limited. Based on genetic information, *R. palustris* possesses the ability to produce pyrroloquinoline quinone (PQQ). PQQ is known to play a crucial role in stimulating plant growth, facilitating phosphorous solubilization, and acting as a reactive oxygen species scavenger. However, it is still uncertain whether growth conditions influence *R. palustris*’s production of PQQ and other characteristics. In the present study, it was found that *R. palustris* exhibited a higher expression of genes related to PQQ synthesis under autotrophic culture conditions as compared to acetate culture conditions. Moreover, similar patterns were observed for phosphorous solubilization and siderophore activity, both of which are recognized to contribute to plant-growth benefits. However, these PNSB culture conditions did not show differences in *Arabidopsis* growth experiments, indicating that there may be other factors influencing plant growth in addition to PQQ content. Furthermore, the endophytic bacterial strains isolated from *Arabidopsis* exhibited differences according to the PNSB culture conditions. These findings imply that, depending on the PNSB’s growing conditions, it may interact with various soil bacteria and facilitate their infiltration into plants.

## 1. Introduction

*Rhodopseudomonas palustris*, a purple non-sulfur bacterium (PNSB), is capable of growing via photoheterotrophic or photoautotrophic metabolism [1]. This PNSB was widely utilized in agriculture, with reports indicating that it has the capacity to enhance the development of numerous crops such as rice [2,3], *Brassica rapa chinensis* (Chinese cabbage) [4], tobacco [5], and *Agaricus bisporus* mushroom [6]. Furthermore, when utilized as a foliar fertilizer, *R. palustris* has been shown to promote the growth of leaves in Chinese dwarf cherry plants [7]. A study also reveals that *R. palustris* does not invade plants like endophytes but rather attaches to plant surfaces [8]. However, we still have a limited understanding of the mechanisms by which they promote plant growth.

Pyrroloquinoline quinone (PQQ), a redox cofactor utilized by glucose, alcohol, and methanol dehydrogenases, is present in microorganisms, plants, and animals [9]. PQQ is also known to play a crucial role in stimulating plant growth via plant-growth-promoting rhizobacteria [10], wherein it can aid in phosphate solubilization [11] or act as a reactive oxygen species scavenger [10]. Additionally, PQQ functions as a vital cofactor in PQQ-dependent glucose dehydrogenase (GDH), which produces gluconic acid to facilitate phosphate solubilization and enhance plant growth [11,12,13]. In addition to its role as a cofactor for GDH, the mere presence of PQQ has been shown to increase the fresh weight and antioxidative capability of cucumbers [10].

The presence of PQQ synthesis genes has been identified in several plant-growth-promoting bacteria, including our previous isolate *Burkholderia seminalis* 869T2 [14], *Pseudomonas fluorescens* B16 [10], and *Rahnella aquatilis* HX2 [15]. These genes include *pqqA*, *pqqB*, *pqqC*, *pqqD*, *pqqE,* and peptidase genes [9]. According to one study, deleting *pqqA* in *Methylbacterium extorquens* AM1 will simply reduce PQQ synthesis [16]. This suggests that there were other genes that could substitute PqqA as precursor peptides to synthesis PQQ [16]. In general, the glutamate and tyrosine in the precursor peptide PqqA would be coupled with a chaperone PqqD and an amino acid linking enzyme PqqE complex during PQQ production [9]. Then the linking amino acids would be cleaved from precursor peptide with peptidases for oxidation and close the indole ring by PqqB and PqqC, respectively [9]. Several peptidases were related to PQQ synthesis, such as PqqF, PqqL, PqqG, PqqH, PqqM, and TldD [10,17,18,19,20]. These peptidases can be classified into four groups by conserved protein domains as coenzyme PQQ biosynthesis probable peptidase PqqF, Zn-dependent peptidase (PqqL), dipeptidyl aminopeptidase/acylaminoacyl peptidase. and metalloprotease TldD (PmbA_TldD superfamily) [21] (Table 1).

*R. palustris* CGA009 is the first strain of this species that has had the whole genomic DNA sequenced [1]. Although *pqqB*, *pqqC*, *pqqD*, and *pqqE* were discovered and annotated in the genome, the absence of the *pqqA* gene raised questions regarding the production of PQQ in *R. palustris* CGA009. We hypothesized that *R. palustris* CGA009 would exhibit the same characteristic in light of the Toyama and Lidstrom [16] findings stating that the *pqqA* gene is not required for PQQ synthesis in *M. extorquens* AM1. According to data from the National Library of Medicine’s National Center for Biotechnology Information (NCBI), the Prokaryotic RefSeq Genome Re-annotation Project recently identified *pqqA* in the genomic DNA of the *R. palustris* CGA009 species. As a result, the puzzle component might be discovered. In this study, we investigated the PQQ production of *R. palustris* CGA009 under both photoautotrophic and photoheterotrophic conditions. Subsequently, we conducted analyses of plant-growth-promoting traits to assess the potential impact on *Arabidopsis* growth. Additionally, based on our observations from lectures in the field of agriculture, some farmers would regularly cultivate photosynthetic bacteria as a method of fertilizing their crops using fish sauces and monosodium glutamate (FsMSG) as the main materials. Therefore, we utilized these circumstances in experiments.

## 2. Results

### 2.1. PQQ Synthesis Genes in Rhodopseudomonas Palustris CGA009 Genome

The PQQ synthesis genes have been well studied for years [9]. The function and amino acid sequence of PqqA, PqqB, PqqC, PqqD, and PqqE were also clarified [17,22,23,24,25]. All of the genes that encode the protein sequences can be found in the *R. palustris* CGA009 genome (Table 1). However, there were numerous other candidates for the peptidase that were related to PQQ production. In the literature, proteins PqqF, PqqL, PqqG, PqqH, PqqM, and TldD were proved to be associated with PQQ production [10,17,18,19,20,26]. Based on the conservation of protein domain families [21], these peptidases in question could be categorized into four distinct groups, namely PQQ_syn_pqqF, PqqL, dipeptidyl aminopeptidase/acylaminoacyl peptidase, and PmbA_TldD superfamily. (Table 1). In the genome of *R. palustris* CGA009, there were three genes belonging to the PqqL family and two genes belonging to the PmbA_TldD superfamily (Table 1). These genomic findings suggest that *R. palustris* CGA009 was capable of producing PQQ.

### 2.2. Transcriptional Levels of PQQ Relative Genes

**Figure 1:**
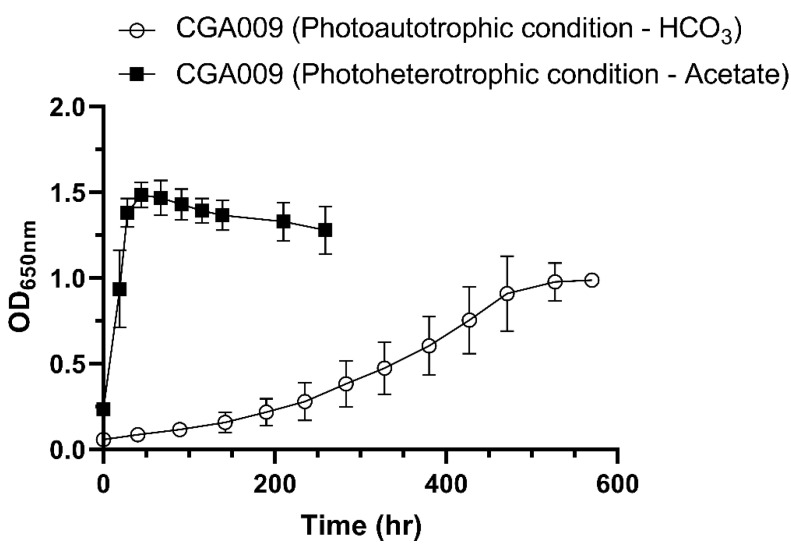
The growth curves of *R. palustris* CGA009 under either photoautotrophic or photoheterotrophic conditions. This image was created using GraphPad Prism version 8.2.1 (https://www.graphpad.com/scientific-software/prism/, accessed on 29 June 2021).

*R. palustris* can grow under either photoautotrophic or photoheterotrophic conditions, using bicarbonate or acetate as carbon sources, respectively (Figure 1). However, the growth under photoautotrophic conditions was slower than that under photoheterotrophic conditions (Figure 1). As a result, samples for each culture condition were taken during the log phase at roughly 0.55 to 0.6 OD_650nm_. In addition, PQQ production was relative to the assimilation of organic compounds, and the amount of PQQ in culture broth was related to the transcription level of PQQ synthesis genes [27]. Therefore, the transcription levels of PQQ production genes can be very different between photoautotrophic and photoheterotrophic culture conditions.

In the bicarbonate medium culture (HCO_3_), the transcriptional levels of PQQ synthesis, PQQ-dependent sugar dehydrogenase, and PQQ-dependent ethanol dehydrogenase genes were significantly higher than that in acetate culture conditions (Table 2). In addition, the high transcriptional levels of ABC transporter genes for sugar, carbohydrate, glycerol 3-phosphate, and branched-chain amino acid might prepare *R. palustris* cells for assimilation sugar or amino acids once present in the culture medium (Supplementary File SI). However, there were no significant differences in the transcription of peptidase genes (Table 2). Therefore, it was not possible to determine which peptidase gene was responsible for PQQ production in *R. palustris* in our results.

### 2.3. Confirmation of PQQ Production in R. palustris CGA009 under Different Culturing Conditions

**Figure 2:**
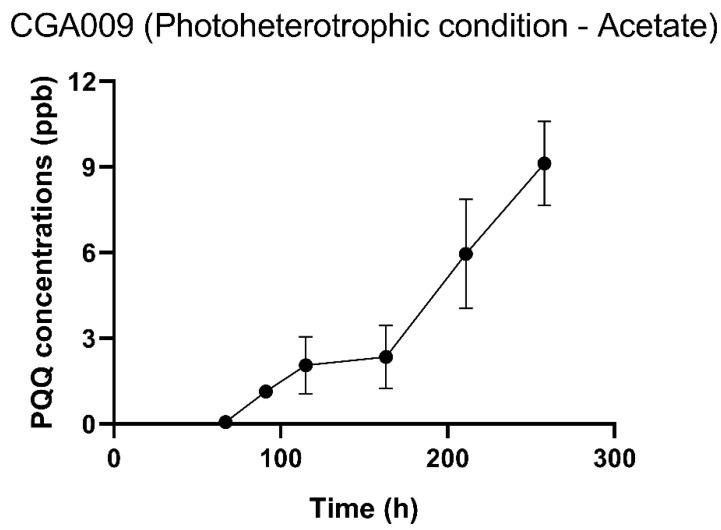
The PQQ production of *R. palustris* CGA009 under heterotrophic conditions with acetate as carbon sources. This image was created using GraphPad Prism version 8.2.1 (https://www.graphpad.com/scientific-software/prism/, accessed on 29 June 2021).

The confirmation of PQQ production was established by either GDH enzymatic assay or liquid chromatography mass spectrometry (LC-MS) analysis. Given the tendency of PQQ to readily form adducts with amino acids and its stability in a PQQ-dependent holoenzyme [28,29]. The GDH enzymatic assay was not suitable for the analysis of amino acid-rich FsMSG samples, and the GDH enzymatic reaction background could have influenced the results obtained for HCO_3_ samples (Table 2). Accordingly, the enzymatic assay was only applicable for the quantification of PQQ concentrations under acetate culture conditions. The PQQ and its adducts for HCO_3_ samples were confirmed by LC-MS analysis (Figure S1). While the transcriptional levels of PQQ genes in the acetate culturing environment of *R. palustris* CGA009 were not as substantial as that under autotrophic conditions (Table 2), the bacterium initiated PQQ production after 70 hours of cultivation (Figure 2). Moreover, the production of PQQ by *R. palustris* CGA009 further increased after 150 hours of culturing (Figure 2). The results also indicated that the quantity of PQQ was below 12 parts per billion (ppb) (Figure 2), which is below the limit of detection in our LC-MS system (Figure S2).

### 2.4. Phosphorus-Solubilizing Activity Assay

**Figure 3:**
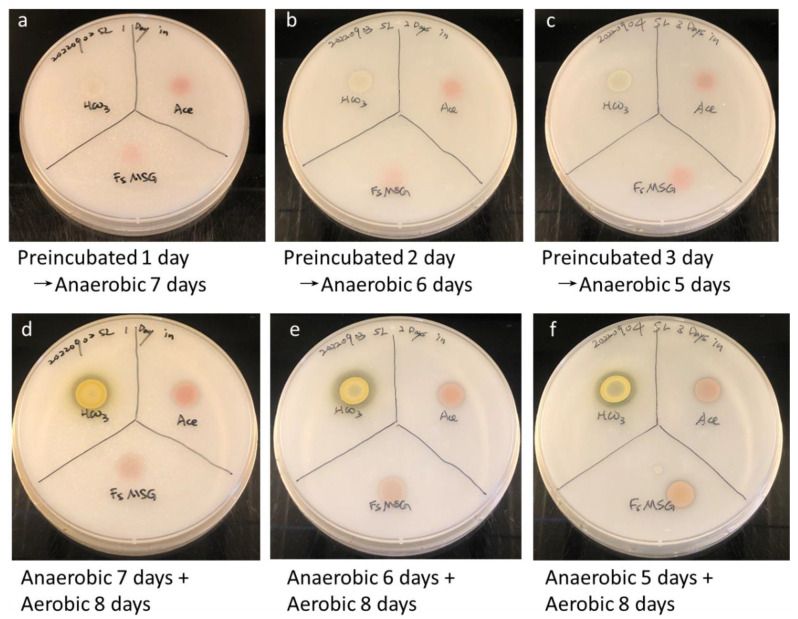
*R. palustris* CGA009 was preincubated in bicarbonate medium (HCO_3_), acetate medium (Ace), or fish sauce with monosodium glutamate medium (FsMSG) for 1 day (**a**), 2 days (**b**), and 3 days (**c**) before testing on phosphate-solubilization assay plates that were incubated in an anaerobic chamber at 35 °C for 7 days (**a**), 6 days (**b**) and 5 days (**c**). Then the plates were moved to an aerobic incubator at 30 °C for 8 days (**d**–**f**). This image was created using Microsoft Office Professional 2019 PowerPoint (https://www.microsoft.com/zh-tw/microsoft-365/p/office-%E5%B0%88%E6%A5%AD%E7%89%88-2019/cfq7ttc0k7c5?activetab=pivot%3aoverviewtab, accessed on 29 June 2021).

Phosphate solubilization represents a crucial mechanism underlying plant-growth promotion [30], wherein certain bacteria can convert insoluble phosphorus into readily available forms for plants. Organic acid production, including the generation of gluconic acid via PQQ-dependent GDH, represents a key means of achieving this conversion [31]. Notably, transcriptional analysis has revealed significantly higher levels of expression for PQQ-dependent genes under bicarbonate culture conditions as compared to acetate culture conditions (Table 2). This could imply that changing culture conditions affect *R. palustris’s* phosphate solubilization activity. Therefore, the *R. palustris* cells were treated with a different medium several days before inoculating on calcium phosphate plates. The treated medium contained either bicarbonate (HCO_3_), acetate (Ace), or fish sauce with monosodium glutamate (FsMSG) as carbon sources. Following the transfer from the HCO_3_ medium to the glucose or FsMSG medium, the colonies of *R. palustris* underwent a distinct color change from red to yellow in dark conditions (Figure S3). Furthermore, extended preculture in bicarbonate conditions resulted in a cleaner zone on assay plates under anaerobic conditions (Figure 3a–c). It was proposed that PQQ synthesis would need oxygen for oxidation [9]. Therefore, the phosphate solubilization activity present under anaerobic conditions could be from the synthesis of PQQ and relative enzymes under microaerobic conditions during preculture in the bicarbonate medium. These results were consistent with transcriptome data (Table 2).

### 2.5. Estimated Siderophore Activity

Several siderophore genes were found to be related to plant-growth promotion. *R. palustris* CGA009 genome harbors multiple genes encoding iron-chelating siderophore proteins. Given the differential gene expression (Supplementary File SI) and metabolic activity between autotrophic and heterotrophic growth modes, we investigated the siderophore activity of this photosynthetic bacterium under varied culture conditions, employing the CAS method [32]. As autotrophic growth was slower than heterotrophic growth (Figure 1 and Table 3), we extended the culture time of the autotrophic group to allow the cell density to reach an OD_650_ of approximately 0.7 before testing. *R. palustris* CGA009 did not exhibit siderophore activity in the medium under heterotrophic growth conditions, but it did exhibit activity under autotrophic growth conditions. These results were consistent with the transcriptome data (Supplementary File SI). In addition, the marked increase in transcription levels of ferritin and siderophore biosynthesis genes (Supplementary File SI) in *R. palustris* suggested a propensity to accumulate iron under autotrophic conditions.

### 2.6. R. palustris CGA009 Effect on Arabidopsis thaliana’s Growth Parameters

**Figure 4:**
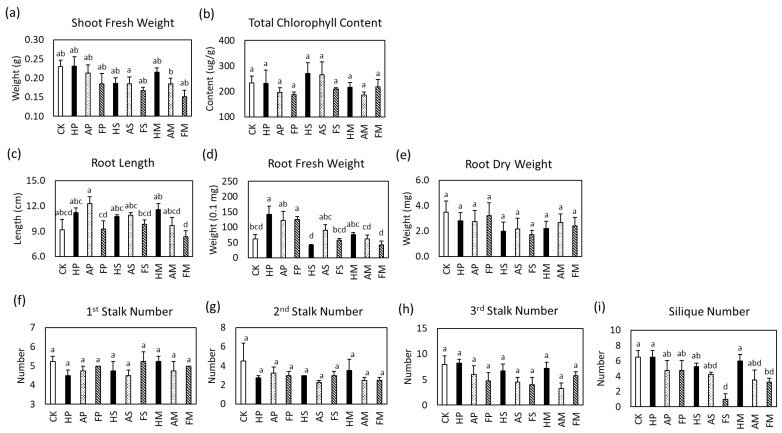
The *Arabidopsis* growths affected by photosynthetic bacteria incubated in different conditions. (**a**) Shoot fresh weight, (**b**) total chlorophyll content, (**c**) root length, (**d**) root fresh and (**e**) dry weight were measured to evaluate the plant vegetative growth; (**f**) The first, (**g**) second, and (**h**) third stalk number and the (**i**) silique number were measured to evaluate the plant reproductive growth. CK, control check; HP, HCO_3_ cell pellet; AP, acetate cell pellet; FP, FsMSG cell pellet; HS, HCO_3_ supernatant; AS, acetate supernatant; FS, FsMSG supernatant; HM, HCO_3_ medium; AM, acetate medium; FM, FsMSG medium. Total chlorophyll content, *n* = 3; the vegetative, *n* = 6–9; the reproductive, *n* = 4. Different letters indicate statistically significant differences at *p* ≤ 0.05. This image was created using Microsoft Office Professional 2019 PowerPoint (https://www.microsoft.com/zh-tw/microsoft-365/p/office-%E5%B0%88%E6%A5%AD%E7%89%88-2019/cfq7ttc0k7c5?activetab=pivot%3aoverviewtab, accessed on 29 June 2021).

Following centrifugation, the bacterial pellets and culture supernatants of CGA009 strains from various culture groups were separated. The pellets and supernatants from the other groups were diluted to the same concentration as the lowest concentration of the autotrophic group and subsequently utilized for *Arabidopsis thaliana* growth experiments. Two weeks after inoculation (2WAI), nine plant-growth parameters were measured to evaluate the effects of inoculation on Arabidopsis growth. These parameters included shoot fresh weight, total chlorophyll content, root length, root fresh weight, root dry weight, and the number of first to third stalks and silique (Figure 4). The primary disparity between the experimental and control groups was observed in the root fresh weight (Figure 4d). Notably, the use of bacteria pellets (HP, AP, and FP) resulted in an increase in root fresh weight irrespective of the culture group, whereas irrigation with culture supernatants alone (HS, AS, and FS) did not yield significant differences with the control group. Although the literature has shown that CGA009 can promote the growth of rice [3], the results above indicate that CGA009 did not have a significant growth-promoting effect on *Arabidopsis*. It is worth noting that the FsMSG media employed in this experiment were prepared using tap water without autoclaving to mimic field conditions (Figure S4).

### 2.7. Identification of Diverse Endophytes in Arabidopsis following R. palustris CGA009 Inoculation

As the phenotypic differences in *Arabidopsis* experiments primarily occurred in root fresh weight (Figure 4d), it was likely that some changes had occurred in the plants. After three days of inoculation, the plants were cleaned with tap water, sterilized with bleach, and their fluids extracted and diluted with sterilized Milli-Q water. The plant extracts were spread onto Lysogeny-Broth (LB) agar plates and incubated at 30 °C for 3 days. Only the control groups did not present any colony on plates (Figures S5b and S6b). Several single colonies were chosen based on their colony morphology and subsequently incubated in LB broth for genomic DNA extraction. The 16S rRNA genes of these bacteria were amplified and sequenced using E8F/U1510R primer pairs, following the procedure described in our previous report [33]. As some colonies were unable to grow individually in LB broth, we were unable to identify every bacterium in our trials. Nevertheless, the identified endophytes in each condition were distinct (Table 4). The majority of the identified species were also reported in various soil samples, with some of these species having been previously characterized as plant-growth-promoting bacteria in other studies, such as *Stenotrophomonas maltophilia* and *Burkholderia anthina* [34,35].

In the previous literature, it was reported that photoautotrophic bacteria subjected to heterotrophic conditions resulted in a reduced proportion of *Burkholderiales* and *Pseudomonas* in the rhizosphere [3,36]. However, our results found that under similar conditions, *Burkholderiales* and *Pseudomonas* were able to enter the plant tissue (Table 4). Whether the reduced rhizosphere microbiota is capable of entering the plant tissue remains to be explored in future studies, and the underlying mechanisms require further investigation to clarify the possibilities.

**Table 4:** The isolated endophytes from the *Arabidopsis thaliana* that were treated with *Rhodopseudomonas palustris* culture cells or supernatants from different culture conditions.

Conditions	Pellets or Supernatant	Endophyte Colony	Phosphorus-Solubilizing Activity	The Best Match with 16S rRNA Gene in BLAST Results (Identity %)	Relative Researches
HCO_3_ medium, microaerobic, light, 30 °C, 17 days	*R. palustris* Pellets	HP1	No	*Stenotrophomonas maltophilia* (99.51%)	Plant-growth-promoting rhizobacterium against stress conditions [34]
		HP2	Yes	*Microbacterium proteolyticum* (99.21%)	Endophytic bacterium isolated from roots of *Halimione portulacoides* [37]
		HP3	Yes	*Paraburkholderia pallidirosea* (97.70%) ^1^	Belong to plant-beneficial environmental groups of bacterium [38]
	Supernatant	HS1	No	*Rhodanobacter lindaniclasticus* (99.17%)	A lindane-degrading bacterium [39]
		HS2	Yes	*Rhodanobacter fulvus* (99.15%)	Biological control activity towards the root-rot plant pathogen *Cylindrocladium spathiphylli* [40]
		HS3	Yes	*Rhodanobacter soli* (93%) ^1^	A soil bacterium from a ginseng field [41]
Acetate medium, microaerobic, light, 30 °C, 8 days	*R. palustris* Pellets	AP1	Yes	*Burkholderia anthina* (99.79%)	Plant-growth-promoting bacteria of sugarcane [35]
	Supernatant	AS1	Yes	*Pseudomonas citronellolis* (95.17%) ^1^	Multi-metal resistant [42]
FsMSG medium, microaerobic, light, 30 °C, 8 days	*R. palustris* Pellets	FP1	Yes	*Achromobacter insuavis* (94.45%) ^1^	Could be isolated from cystic fibrosis patients [43]
		FP2	Yes	*Achromobacter insuavis* (99.56%)	
		FP3	Yes	*Achromobacter insuavis* (99.86%)	
	Supernatant	FS1	No	*Paraburkholderia kururiensis* (99.58)	A trichloroethylene-degrading bacterium [44]
		FS2	No	*Ferrovibrio xuzhouensis* (99.49%)	A cyhalothrin-degrading bacterium [45]
		FS3	Yes	*Amycolatopsis rhabdoformis* (98.81%)	A soil bacterium from a tropical forest [46]

^1^ A sequence identity of less than 98.7% suggested that the bacterium might not belong to the expected species.

## 3. Discussion

*R. palustris* is a purple non-sulfur bacterium that can be isolated from environmental water bodies and rice fields [4,47]. Since it was found that spraying this bacterium on crops can improve plant growth, and considering its low cultivation cost [48], it has been widely accepted by farmers in recent years. There are also studies exploring the interaction between phototrophic bacteria and plants, such as their ability to produce plant-growth-promoting substances [49]. The main contributions of phototrophic bacteria to plants include aiding in nitrogen fixation, solubilizing phosphate, and increasing the content of soluble sugars and secondary metabolites [50,51,52]. Additional plant-growth-promoting mechanisms of PNSB can be explored in the comprehensive review [49]. It is noteworthy that while *R. palustris* is capable of producing indole-3-acetic acid (IAA), the observed increase in IAA levels within Chinese cabbage leaves appears to be influenced by other unidentified factors [8]. It has been reported that rhizosphere irrigation with *Rhodopseudomonas* sp. can alter the microbial community in the soil [3,52]. However, the primary factor responsible has not yet been identified.

Despite the presence of genes encoding PQQ-dependent glucose dehydrogenase [53] and PQQ synthesis genes (Table 1) in *R. palustris* CGA009, this strain did not solubilize inorganic phosphorus from insoluble substances in a previous report [53]. Other PNSB strains, however, have been noted to exhibit minimal phosphate solubilization activity in various growth mediums [54]. In this study, two trophic culture conditions were employed to examine the transcriptional levels of PQQ synthesis and PQQ-dependent enzyme genes. These genes exhibited a higher transcriptional level under autotrophic conditions than under acetate culture conditions (Table 2). Although the PQQ production under autotrophic conditions was not quantified using the LC-MS system, the detectable amount of PQQ indicates that the concentration was above the detection limit of 60 ppb, which was higher than that observed under acetate culture conditions. (Figures S1, S2, and Figure 2). Additional PQQ and amino acid adducts were also detected in the medium under autotrophic conditions (Figure S1). Previous studies have demonstrated that the addition of synthetic PQQ can enhance the fresh and dry weight of *Arabidopsis* [10]. However, the growth-stimulating characteristics were not observed in the plant-growth experiments (Figure 4). Additionally, PQQ-adducts with amino acids have been reported to stimulate the growth of microorganisms such as *E. coli* and *Pseudomonas* [28,55]. It is reasonable to suggest that PQQ-adducts with amino acids could stimulate other soil bacteria. Notably, three *Rhodanobacter* species were isolated only in the HCO_3_-supernatant experiment group (Table 4), suggesting that the HCO_3_-supernatant may stimulate *Rhodanobacter* to invade plants. These results suggest that although the supernatant under autotrophic culture conditions may contain higher levels of PQQ than that under heterotrophic culture conditions, there may be other factors that influence plant growth.

Based on the consistent results of phosphorus-solubilizing activities (Figure 3) and siderophore activities (Table 3), it was expected that PNSB pellets would have a better plant-growth-promoting effect under autotrophic conditions. However, the results of the plant experiment did not support this hypothesis. Although the biochemical assays yielded disparate outcomes, the three groups of PNSB culture conditions did not exhibit significant differences in plant-growth experiments (Figure 4). Nevertheless, the experimental groups treated with PNSB pellets exhibited higher root fresh weights compared to the control group (Figure 4d). Following the isolation of endophytes, diverse bacterial species were identified except in the control group (Figures S5b and S6b, and Table 4). However, since the juices of three plant samples were pooled for each treatment group, the isolated endophytes may not be present in every sample within the same group. Despite this, the experimental groups still exhibited distinct endophytic colonies, particularly in the FsMSG-pellet group where three isolated bacteria similar to *Achromobacter insuavis* were identified (Table 4). These findings suggest that PNSB interacts with different rhizobacteria depending on the culture conditions.

The majority of the isolated bacterial species in all experiment groups were not previously reported to be associated with plant-growth promotion (Table 4). However, some of the isolated bacterial species have been reported as plant-growth-promoting rhizobacteria, such as *Stenotrophomonas maltophilia* HP1 [34] and *Burkholderia anthina* AP1 [35] (Table 4). Therefore, the results suggest that PNSB pellets prepared under HCO_3_-culture or acetate-culture have the potential to induce plant-growth-promoting rhizobacteria to colonize plants.

## 4. Materials and Methods

### 4.1. Culture Conditions

*R. palustris* CGA009 was purchased from the American Type Culture Collection (ATCC) and cultivated using *Rhodospirillaceae* media under the photoheterotrophic conditions detailed below. For long-term storage, the bacteria culture was supplemented with 10% glycerol and maintained at −80 °C. For experiments, *R. palustris* CGA009 was cultured in an FsMSG medium or *Rhodospirillaceae* media with modifications for certain conditions [56]. In general, 1 L of medium contained 0.5 g of KH_2_PO_4_, 0.5 g of K_2_HPO_4_, 0.2 g of MgSO_4_·7H_2_O, 0.4 g of NaCl, 0.05 g of CaCl_2_·2H_2_O, 0.05 g of Fe-citrate, 1 g of (NH_4_)_2_SO_4_, 70 μg of ZnCl_2_, 100 μg of MnCl_2_·4H_2_O, 20 μg of CuCl_2_·2H_2_O, 200 μg of CoCl_2_·6H_2_O, 20 μg of NiCl_2_·6H_2_O, 40 μg of NaMoO_4_·2H_2_O, and 60 μg of H_3_BO_3_. The autotrophic medium containing HCO_3_^−^ as the carbon source was supplemented with 11.26 g of Na_2_S_2_O·5H_2_O per liter as an electron donor. After autoclaving, 50 mL of filter-sterilized NaHCO_3_ solution containing 1.68 g of NaHCO_3_ was added. For the heterotrophic culture condition, 1 g of CH_3_COONa was added to 1 L of medium and autoclaved. The FsMSG medium was prepared by adding 2 g of fish sauce (Thai Pure, Qua-Quality, Taipei City, Taiwan) and 2 g of monosodium L-glutamate (Vedan, Taichung City, Taiwan) to 1 L with tap water, followed by sterilization using a 0.22 μm filter, unless otherwise indicated. Prior to inoculation under specific culture conditions, the cells were precultured in the *Rhodospirillaceae* medium containing 1 g/L of sodium acetate and 0.2 g/L of yeast extract for 3 days. After washing twice with autoclaved Milli-Q water, the cells were inoculated into the conditioned medium, except for the acetate and FsMSG culture conditions. *R. palustris* cells were cultured in tubes filled with the medium and incubated at 30 °C under illumination with a 100 W tungsten filament lamp at 3000 lx. Bacterial growth was measured by monitoring the optical density at 650 nm (GeneQuant 1300, GE Healthcare, Little Chalfont, Buckinghamshire, UK).

### 4.2. Transcriptome Analysis

Total RNA was extracted from *R. palustris* strains that had been incubated until the log phase, at approximately 0.55 to 0.6 OD_650nm_, for each culture condition. Reverse transcription and DNA sequencing (Illumina) were conducted by Welgene Biotech Co., Ltd. (Taipei, Taiwan). Total RNA was extracted using Isol-RNA Lysis Reagent (5 PRIME, Hamburg, Germany) according to the manufacturer’s instructions. The procedure was the same as that in our previous report [57]. The StringTie (StringTie v2.1.4) based protocol [58] was used to calculate differential gene expression between the HCO_3_-culture and acetate-culture strains.

### 4.3. Phosphorus-Solubilizing Activity Assay

The ability of *R. palustris* to solubilize inorganic phosphorus was observed by the halo formed after culturing on modified agar plates containing Ca_3_(PO_4_)_2_. In general, 1 L of medium contained 15 g of agar, 5 g of Ca_3_(PO_4_)_2_, 0.2 g of KCl, 0.2 g of MgSO_4_·7H_2_O, 0.4 g of NaCl, 0.05 g of CaCl_2_·2H_2_O, 0.05 g of Fe-citrate, 1 g of (NH_4_)_2_SO_4_, 70 μg of ZnCl_2_, 100 μg of MnCl_2_·4H_2_O, 20 μg of CuCl_2_·2H_2_O, 200 μg of CoCl_2_·6H_2_O, 20 μg of NiCl_2_·6H_2_O, 40 μg of NaMoO_4_·2H_2_O, and 60 μg of H_3_BO_3_. After adding 5 g of glucose, the pH was adjusted to 7.5 using 5 N NaOH. In cases where indicated, 1 g of sodium acetate was used instead of glucose. The FsMSG agar plate supplemented with inorganic phosphorus was prepared by adding 5 g of Ca_3_(PO_4_)_2_ and 15 g of agar to 1 L of FsMSG medium. After cultivation of *R. palustris* CGA009 under various conditions, 20 μL of each culture was inoculated onto inorganic phosphorus agar plates and subsequently incubated either under aerobic conditions in an incubator at 30 °C or in an anaerobic chamber (Coy Laboratory Products, Grass Lake, MI, USA) at 35 °C.

### 4.4. Siderophore Estimation Assay

The siderophore activity was determined using the universal chrome azurol S (CAS) assay [32] by the modified microplate method [59]. To prepare solutions for the CAS assay, 36 mg of hexadecyl trimethyl ammonium bromide (HDTMA) was dissolved in 10 mL of deionized water to obtain a 10 mM HDTMA solution. Subsequently, 20 μL of 5 N HCl was added to 10 mL of deionized water to obtain a 10 mM HCl solution. Next, 2.7 mg of FeCl_3_·6H_2_O was dissolved in 10 mL of the 10 mM HCl solution to obtain a 1 mM FeCl_3_ solution. Finally, 12 mg of chrome azurol S (CAS) was dissolved in 10 mL of deionized water to obtain a 2 mM CAS solution. Finally, the CAS assay solution was prepared as follows: 0.15 mL of 1 mM FeCl_3_ solution and 0.75 mL of 2 mM CAS solution were mixed and added to 0.6 mL of 10 mM HDTMA solution, followed by the addition of 1.9 mL of deionized water. The culture supernatant was obtained by centrifugation and filtration through a 0.22 μm filter. A total of 100 μL of each filtered sample was mixed with 100 μL of CAS assay solution in a 96-well microplate, followed by thorough mixing. After incubating the mixture for 24 h at room temperature in the dark, the absorbance at 630 nm was measured using a microplate reader (Paradigm, Beckman Coulter Inc., California, USA). The percent siderophore unit (psu) was calculated based on the method previously described in the literature [59].

### 4.5. PQQ Extraction for LC-MS Analysis

The extraction of PQQ was performed using a previously established protocol with certain modifications [60]. Using a vacuum concentrator (MICRO-CENVAC, N-Biotek, Korea), 0.6 mL of *R. palustris* culture supernatant was reduced to 0.4 mL by heating. The concentrated sample was mixed well with 0.2 mL of BHP solution (benzalkonium chloride:n-hexane:n-pentanol, *w*/*v*/*v* = 0.5:9.5:1) and shaken for 10 min. After standing for 1 min, the upper layer of the BHP solution was carefully transferred to a new 1.5 mL centrifuge tube that contained 0.4 mL of 15% NaCl. The resulting mixture was shaken for 10 min and then left to stand for 1 min to allow for phase separation. In the final step, the lower layer solution was directly collected using a micropipette, while the upper layer BHP solution was recovered. The lower layer solution was then stored at 4 °C until it was analyzed using LC-MS.

### 4.6. LC-MS Method

The determination of PQQ and its adducts was carried out following previously published methods [61,62]. In detail, the analysis of PQQ was performed using the negative ion electrospray ionization (ESI) mode on a Thermo Ultimate 3000 ultra-performance liquid chromatography (UPLC) system (Dionex/Thermo Fisher Scientific, Idstein, Germany) coupled with an amaZon speed mass spectrometer. The obtained data was analyzed using the Compass Data Analysis software, Vision 4.0 (Bruker, Billerica, MA, USA). The samples were subjected to separation on a C18 column (150 mm × 2.1 mm, 3 μm, GL Sciences, Inc., Torrance, CA, USA) using a mobile phase consisting of 10 mM dibutylammonium acetate as mobile phase A and acetonitrile as mobile phase B. The following liquid chromatography gradient was employed: 0–6 min with 70% mobile phase A, followed by a decrease of solvent A to 10% over 6–12 min, and finally, 10 min of equilibration with initial conditions before the next injection, using a flow rate of 200 μL/min. The DAD chromatogram was monitored for absorbance at 249, 280, and 422 nm. The mass spectrometer was operated in multiple reaction monitoring (MRM) mode, with m/z 329 selected as the precursor ion of PQQ and m/z 241 and m/z 285 as the product ions of PQQ. The electrospray ionization parameters were set as follows: a dry gas flow rate of 9.0 L/min, a nebulizing gas flow rate of 40 psi, a dry temperature of 250 °C, and an ionization voltage of −4500 V.

### 4.7. Enzymatic PQQ Determination

#### 4.7.1. Preparation of *E. coli* Membrane Fractions

The cell membrane fraction of *E. coli* DH5α contains apo-PQQ-dependent glucose dehydrogenase; however, *E. coli* does not produce PQQ [63]. Therefore, it can be employed to determine PQQ concentrations [64,65]. *E. coli* was precultured in 3 mL of the Lysogeny broth (LB) medium overnight. Then 1.4 mL of preculture *E. coli* was inoculated into each of two containers of 300-mL LB medium and shaken (150 rpm) at 37 °C. The cells were harvested by centrifugation at 6000× *g* for 10 min at 4 °C when OD_600_ reached 0.6. The cell pellets were washed twice with 22 mL of phosphate-buffered saline (50 mM PBS; pH 7.0) and by centrifugation at 6000× *g* for 10 min at 4 °C. The pellets were suspended in 10 mL of PBS, and total proteins were extracted by sonication for 300 cycles of 3 s each with a 6-s pause in ice and water. The cell debris was removed by centrifugation at 8000× *g* for 5 min at 4 °C. The membrane fraction of total protein was collected by ultracentrifugation at 68,000× *g* for 1 h at 4 °C (Optima L-100K, Beckman, Brea, CA, USA). The pellet of the membrane was submerged and washed gently with 0.2 mL PBS. After removing the PBS, the membrane pellet was resuspended and dissolved in 0.8 mL PBS with 2 mM of CaCl_2_. The suspended membrane was frozen at −20 °C. After thawing the suspended membrane, the supernatant of the membrane fraction was collected by centrifugation at 8000× *g* for 3 min at 4 °C and used to determine PQQ concentrations. After three freeze-thaw cycles, the PQQ-dependent glucose dehydrogenase in the membrane fraction maintained its activity (Table S1).

#### 4.7.2. PQQ Bioassays

The PQQ-dependent GDH enzyme activity was measured in the presence of 41 mM of phosphate-buffered saline (pH 7.0), 2 mM of phenazine methosulfate (PMS), 0.06 mM of 2,6-dichlorophenol indophenol (DCPIP), 0.5 mM of CaCl_2_, 4 mM of NaN_3_, and 33 mM of glucose. In general, the *E. coli* membrane fraction described in the above section was diluted to 80 μg/mL with a 50 mM PBS (pH 7.0) buffer that contained 1 mM of CaCl_2_. Reconstitution of PQQ-dependent glucose dehydrogenase was carried out by combining 0.05 mL of the diluted *E. coli* membrane fraction with 0.05 mL of either sample or PQQ standard solutions, followed by incubation at room temperature for 10 min. The reaction buffer was then supplemented with DCPIP prior to mixing with the reconstituted PQQ-dependent glucose dehydrogenase by pipetting. The enzyme mixture (0.95 mL) was added to a cuvette containing 0.05 mL of 660 mM glucose and mixed thoroughly by pipetting. The changes in OD_600nm_ were recorded for a duration of 2 min, and the enzyme activity was determined as the change in OD_600nm_ per minute.

### 4.8. Plant’s Growth Condition

*Arabidopsis thaliana* ecotype Columbia (Col-0) was provided by Chieh-Chen Huang and taken care of as previously described [66]. Briefly, the surface-sterilizing seeds were kept under 4 °C for at least 4 days for seed vernalization and then germinated in plug cells. The inoculation treatments were carried out when the seedlings reached the growth stage of 4 to 6 rosette leaves emergence, which corresponds to the principal growth stage of *Arabidopsis* 1.04 to 1.06 [67]. After inoculation, seedlings were transplanted into pots (diameter: 6.2 cm; depth: 8 cm) and grown in either a plant-growth chamber or room with controlled conditions: 21 ± 2 °C, relative humidity (RH) 40 ± 5%, 14/10 day-night light period and light density 90–120 μmol/m^2^/s. All the seedlings were taken care of by the same watering frequency, and no fertilizer was used in this experiment. The evaluation of the plant’s vegetative or reproductive growth at two weeks after inoculation (2WAI) was performed by measuring a total of eight phenotypic parameters: shoot fresh weight; root length; root fresh weight; root dry weight; first, second, and third stalk quantifications; and silique quantification.

### 4.9. Chlorophyll Content Measurements

The N,N-dimethylformamide (DMF) method previously described [68] was employed in conjunction with 99.8% DMF (cat. 0425-3250; Showa, Japan) to extract chlorophyll. The total chlorophyll content was calculated using the formula: Chl_total_ = 7.12 × A664 + 18.12 × A647.

### 4.10. Isolation and Identification of Endophytes

Three *Arabidopsis thaliana* plants that had been inoculated for 3 days were selected for each experimental group. The plants were washed with tap water and subsequently transferred to a 50 mL centrifuge tube. They were then subjected to surface sterilization by adding 40 mL of a solution containing 10% bleach and 0.1% Tween 20 surfactant, followed by horizontal shaking for 6 min. The bleach solution was discarded under sterile conditions, and the plants were washed four to five times with sterile ddH_2_O by shaking for 30 s each time. To confirm surface sterilization, 20 μL of sterile distilled water from the final wash was cultured on LB plates (Figures S5a and S6a). The three *Arabidopsis* plants were ground into a juice using a mortar and pestle, and the resulting juice was collected in an Eppendorf tube. The juice was diluted 1, 10, 100, 1000, and 10,000 times and spread on LB plates for observation (Figures S5 and S6). The 16S rRNA genes of the endophytic isolates, which could be cultured separately in LB broth, were amplified using E8F/U1510R primer pairs for taxonomic analysis, as previously described [33].

### 4.11. Statistic

In this study, the Shapiro-Wilk test and Levene’s test were carried out to check the normal distribution of variables and the homoscedasticity of the plant experiment data. Either a parametric one-way ANOVA (analysis of variance) with Tukey’s post hoc HSD (honest significant difference) test or a non-parametric Kruskal-Wallis test with Dunn’s post hoc test was then applied for statistical significance. The analysis was performed using the Real Statistics Resource Pack software (release 251 7.7.1), copyright (2013–2021) Charles Zaiontz. www.real-statistics.com. At least three independent biological replicates were tested for all experiments unless otherwise stated. Data points are shown mainly in mean ± SEM, and the statistically significant differences between samples were indicated by different letters (at *p* ≤ 0.05) or asterisks (* *p* ≤ 0.05, ** *p* ≤ 0.01, *** *p* ≤ 0.005, and **** *p* ≤ 0.001); otherwise, they were insignificant.

## 5. Conclusions

In this study, PQQ production in *R. palustris* CGA009 was confirmed under both HCO_3_-culture and acetate-culture conditions. Although the PQQ production, phosphorus-solubilizing activities, and siderophore activities were higher in the HCO_3_ culture than in the acetate culture, both culture conditions increased the root fresh weights in the plant experiments, similar to the FsMSG culture. However, the endophyte species isolated from each group of plants were distinct, suggesting that the bacteria present in the soil may also play a key role in using PNSB as a plant-growth-promoting bacterium. The findings also imply that PNSB may promote the invasion of soil bacteria into plants. However, further investigation is required to elucidate the potential interaction mechanisms.

## Figures and Tables

**Table 1:** The possible PQQ synthesis genes in *R. palustris* CGA009.

PQQ Synthesis Related Genes	Conserved Protein Domain Family	References for PQQ Synthesis	Locus Tag in CGA009 (NCBI Reference Sequence: NC_005296.1) ^1^
*pqqA*	PQQ_syn_pqqA (TIGR02107)	[17,22,23]	TX73_RS09945
*pqqB*	PRK05184	[17,24,25]	TX73_RS09950
*pqqC*	PRK05157	[17]	TX73_RS09955
*pqqD*	PqqD Superfamily (cl05126)	[17]	TX73_RS09960
*pqqE*	PRK05301	[17,25]	TX73_RS09965
*Peptidases*			
*pqqF*	PQQ_syn_pqqF (TIGR02110)	[17]	None
*pqqL*	PqqL (COG0612)	[18]	TX73_RS04330, TX73_RS22305, TX73_RS22310
*pqqG*	PqqL (COG0612)	[20]	TX73_RS04330, TX73_RS22305, TX73_RS22310
*pqqH*	DAP2 (COG1506) ^2^	[26]	none
*pqqM*	DAP2 (COG1506) ^2^	[10]	none
*TldD*	PmbA_TldD Superfamily (cl19356)	[19]	TX73_RS04255, TX73_RS05895

^1^ These *R. palustris* CGA009 genes were matched to the conserved protein domain family from the PQQ-synthesis-related genes. ^2^ DAP2 indicates Dipeptidyl aminopeptidase/acylaminoacyl peptidase.

**Table 2:** The gene transcriptional levels (transcripts per million, TPM) of *Rhodopseudomonas palustris* CGA009 when using bicarbonate (HCO_3_) or acetate as carbon sources. The samples were collected when the cell densities reached around 0.55 to 0.6 at OD_650nm_.

Gene Name ^1^	Gene Description ^2^	HCO_3_ (TPM)	Acetate (TPM)	log2 Ratio	*p* Value
**PQQ-dependent enzyme genes**					
TX73_RS03805	PQQ_dependent_sugar_dehydrogenase	136.1	3.3	5.33	0.007
TX73_RS16265	PQQ_dependent_dehydrogenase__methanol_ethanol_family	298.7	2.5	6.81	0.006
TX73_RS22090	PQQ_dependent_sugar_dehydrogenase	18.0	8.5	1.06	0.429
**PQQ synthesis genes**					
*pqqA*	pyrroloquinoline_quinone_precursor_peptide_PqqA	2523.6	160.9	3.97	0.039
*pqqB*	pyrroloquinoline_quinone_biosynthesis_protein_PqqB	159.7	6.9	4.51	0.016
*pqqC*	pyrroloquinoline_quinone_synthase_PqqC	69.3	5.0	3.78	0.016
*pqqD*	pyrroloquinoline_quinone_biosynthesis_peptide_chaperone_PqqD	30.5	1.7	4.12	0.004
*pqqE*	pyrroloquinoline_quinone_biosynthesis_protein_PqqE	20.9	1.6	3.65	0.013
TX73_RS20405	PqqD_family_protein	38.3	7.3	2.39	0.066
**Peptidase genes** ^3^					
TX73_RS04330	Predicted Zn-dependent peptidase (PqqL)	20.7	35.2	−0.76	0.608
TX73_RS22305	Predicted Zn-dependent peptidase (PqqL)	12.1	18.2	−0.59	0.669
TX73_RS22310	Predicted Zn-dependent peptidase (PqqL)	13.3	21.6	−0.69	0.620
*tldD*	PmbA_TldD Superfamily	116.8	149.9	−0.35	0.847
TX73_RS05895	PmbA_TldD Superfamily	15.0	39.7	−1.39	0.357

^1^ The gene names were adapted from RefSeq in the National Center for Biotechnology Information (NCBI). ^2^ The gene descriptions were adapted from RefSeq in the National Center for Biotechnology Information (NCBI), except the peptidase genes. ^3^ The gene descriptions of the peptidase genes were adapted from the Conserved Domain Database (CDD).

**Table 3:** The estimated siderophore activity of *Rhodopseudomonas palustris* CGA009 under different culture conditions. The percent siderophore unit data were presented as the mean and standard deviation of three measurements.

Medium	Culture Condition	Culture Period (Day)	Final OD650	Percent Siderophore Unit
HCO_3_	Photoautotrophic	17	0.745	28.9 ± 4
Acetate	Photoheterotrophic	8	1.84	0
FsMSG	photoheterotrophic	8	4.19	0

## Data Availability

All of the sequencing data were submitted to NCBI (accession numbers of 16S rRNA: OR437490-OR437503; accession numbers of NGS sequence read: SRR25637384 and SRR25637383).

## Supplementary Materials

The supporting information can be downloaded at: https://www.mdpi.com/article/10.3390/ijms241814080/s1.
