# Supplementary material for: Characterization of the Pyrroloquinoline Quinone Producing Rhodopseudomonas palustris as a Plant Growth-Promoting Bacterium under Photoautotrophic and Photoheterotrophic Culture Conditions
Source: Int J Mol Sci. 2023 Sep 14;24(18):14080. doi: 10.3390/ijms241814080 (PMC10531626; doi:10.3390/ijms241814080)
Supplement: Supplementary file 1 [file ijms-24-14080-s001.zip › Supplementary File SII.pdf]

Characterization of the PQQ producing *Rhodopseudomonas palustris* as a plant growth-promoting bacterium under photoautotrophic and photoheterotrophic culture conditions

Shou-Chen Lo[1], Shang-Yieng Tsai[1], Wei-Hsiang Chang[1], I-Chen Wu[1], Nga-Lai Sou[2], Shih-Hsun Walter Hung[1,3], En-Pei Isabel Chiang[2,4,5], Dony Chacko Mathew[6] and Chieh-Chen Huang[1,4,5*]

1 Department of Life Sciences, National Chung Hsing University, Taichung, Taiwan; scl@dragon.nchu.edu.tw (S.-C.L.); seiken.public@gmail.com (S.-Y.T.); ben0306jamin@gmail.com (W.-H. C.); kyle172224@gmail.com (I.-C. W.); walter030170@gmail.com (S.-H.W.H.); cchuang@dragon.nchu.edu.tw (C.-C.H.)
2 Department of Food Science and Biotechnology, National Chung Hsing University, Taichung, Taiwan; looksusan2013@gmail.com (N.-L.S.); chiangisabel@nchu.edu.tw (E.-P.I.C.)
3 Institute of Plant and Microbial Biology, Academia Sinica, Taipei, Taiwan
4 Program in Microbial Genomics, National Chung Hsing University, Taichung, Taiwan
5 Innovation and Development Center of Sustainable Agriculture, National Chung Hsing University, Taichung, Taiwan
6 Washington High School, Taichung, Taiwan; dcmonline4u@yahoo.co.in (D.C.M.)
* Correspondence: cchuang@dragon.nchu.edu.tw

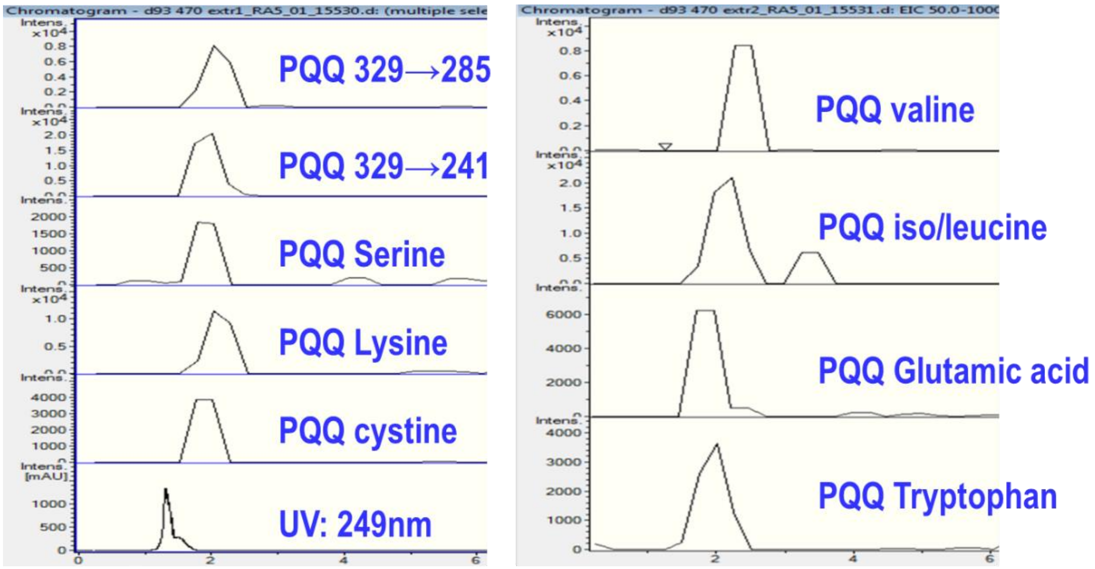

Figure S1. LC-MS results of PQQ and its adducts.

LC-MS analysis was performed to detect PQQ and its adducts in *R. palustris* CGA009 after 470 hours of growth under autotrophic conditions. This image was created by using Compass Data Analysis software, Vision 4.0 (Bruker, Billerica, MA, USA) and Microsoft Office Professional 2019 PowerPoint (https://www.microsoft.com/zh-tw/microsoft-365/p/office-%E5%B0%88%E6%A5%AD%E7%89%88-2019/cfq7ttc0k7c5?activetab=pivot%3aoverviewtab).

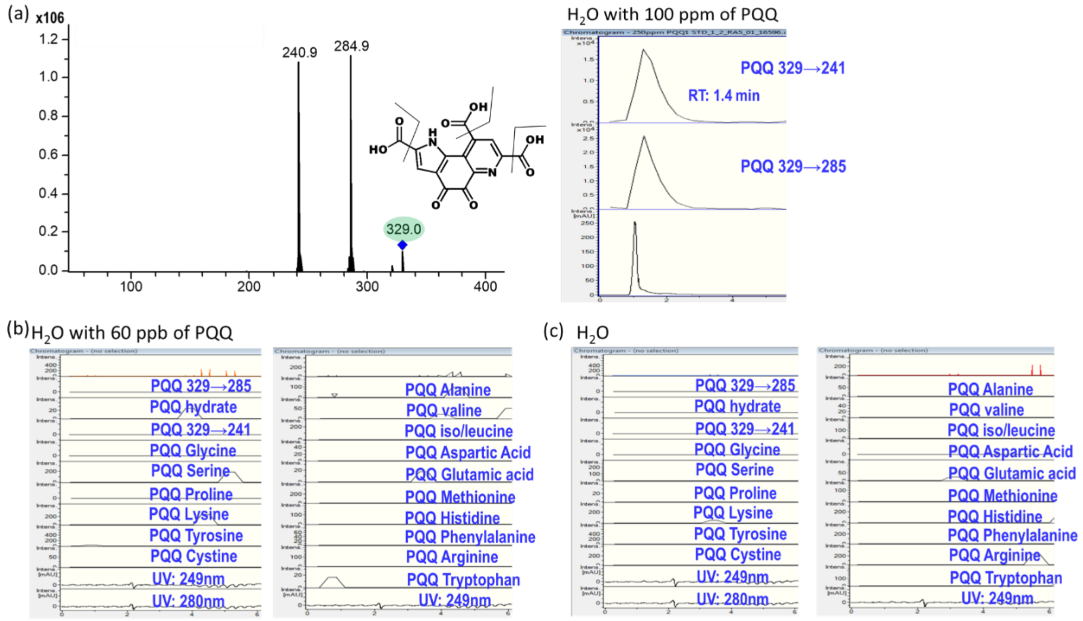

Figure S2. The molecular masses of PQQ as determined by LC-MS.

The precursor ion was at m/z 329; observed fragmentation patterns of the product ions were at m/z 241 and m/z 285 (a). In H_2O samples, PQQ and its adducts were not detected at concentrations below 60 ppb by LC-MS (b, c). This image was created by using Compass Data Analysis software, Vision 4.0 (Bruker, Billerica, MA, USA) and Microsoft Office Professional 2019 PowerPoint (https://www.microsoft.com/zh-tw/microsoft-365/p/office-%E5%B0%88%E6%A5%AD%E7%89%88-2019/cfq7ttc0k7c5?activetab=pivot%3aoverviewtab).

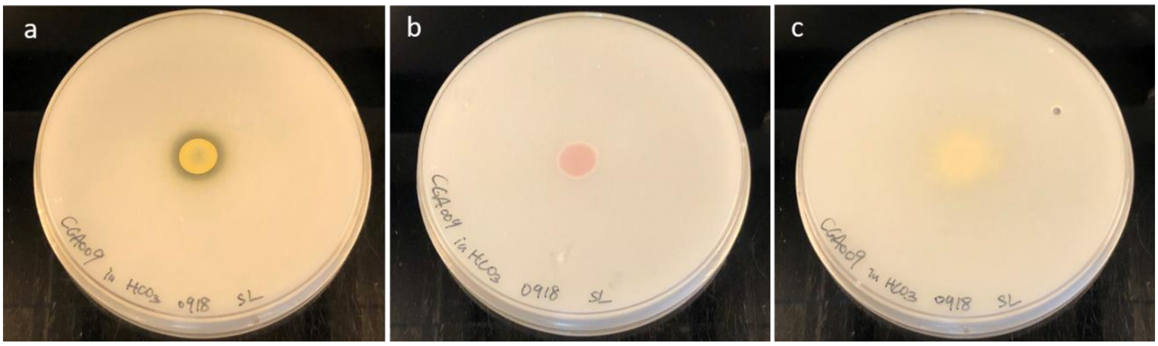

Figure S3. Colonies of *R. palustris* CGA009 pre-cultured under autotrophic conditions and subsequently cultured on different growth media.

The *R. palustris* CGA009 was preincubated in bicarbonate medium (HCO_3) for 17 days and then inoculated on plates contained glucose (a), sodium acetate (b) and fish sauce with monosodium glutamate (FsMSG) media (c). The plates were incubated at 30ºC under aerobic conditions for 8 days. This image was created by using Microsoft Office Professional 2019 PowerPoint (https://www.microsoft.com/zh-tw/microsoft-365/p/office-%E5%B0%88%E6%A5%AD%E7%89%88-2019/cfq7ttc0k7c5?activetab=pivot%3aoverviewtab).

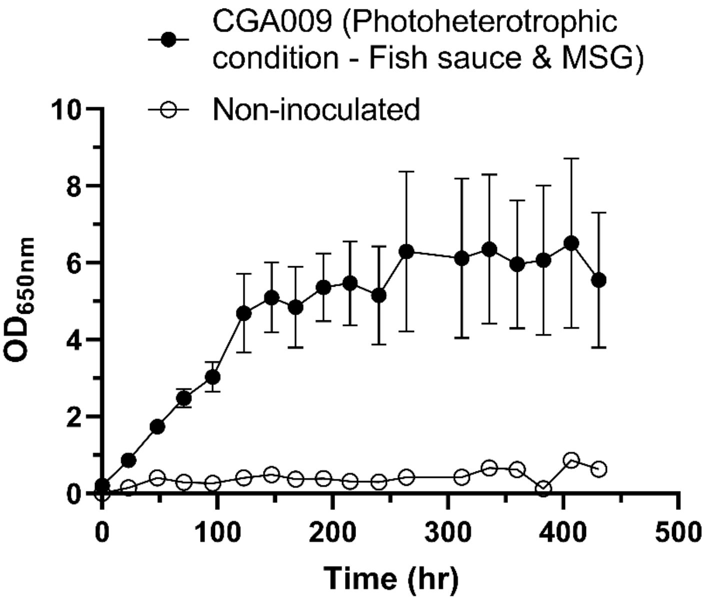

Figure S4. The non-inoculated control and the growth curves of *R. palustris* CGA009 under photoheterotrophic conditions with fish sauce and MSG as carbon sources.
The media were prepared with tap water without autoclaving. This image was created by using GraphPad Prism version 8.2.1 (https://www.graphpad.com/scientific-software/prism/).

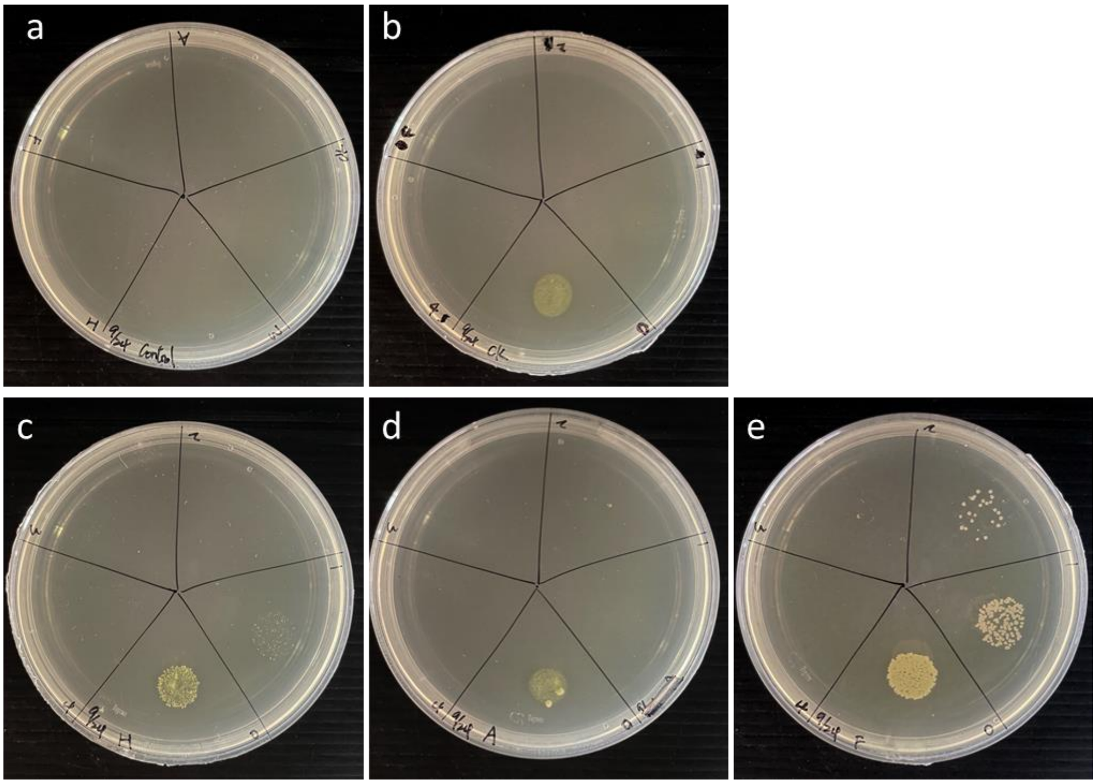

Figure S5. The colonies from pellet inoculated-*Arabidopsis* plant juices on LB plates.
The LB plates were cultured with sterile distilled water obtained from the final wash of samples (a), *Arabidopsis* plant juices without inoculation (b), HCO3-pellet inoculation (c), Acetate-pellet inoculation (d) and FsMSG-pellet inoculation (e). This image was created by using Microsoft Office Professional 2019 PowerPoint (https://www.microsoft.com/zh-tw/microsoft-365/p/office-%E5%B0%88%E6%A5%AD%E7%89%88-2019/cfq7ttc0k7c5?activetab=pivot%3aoverviewtab).

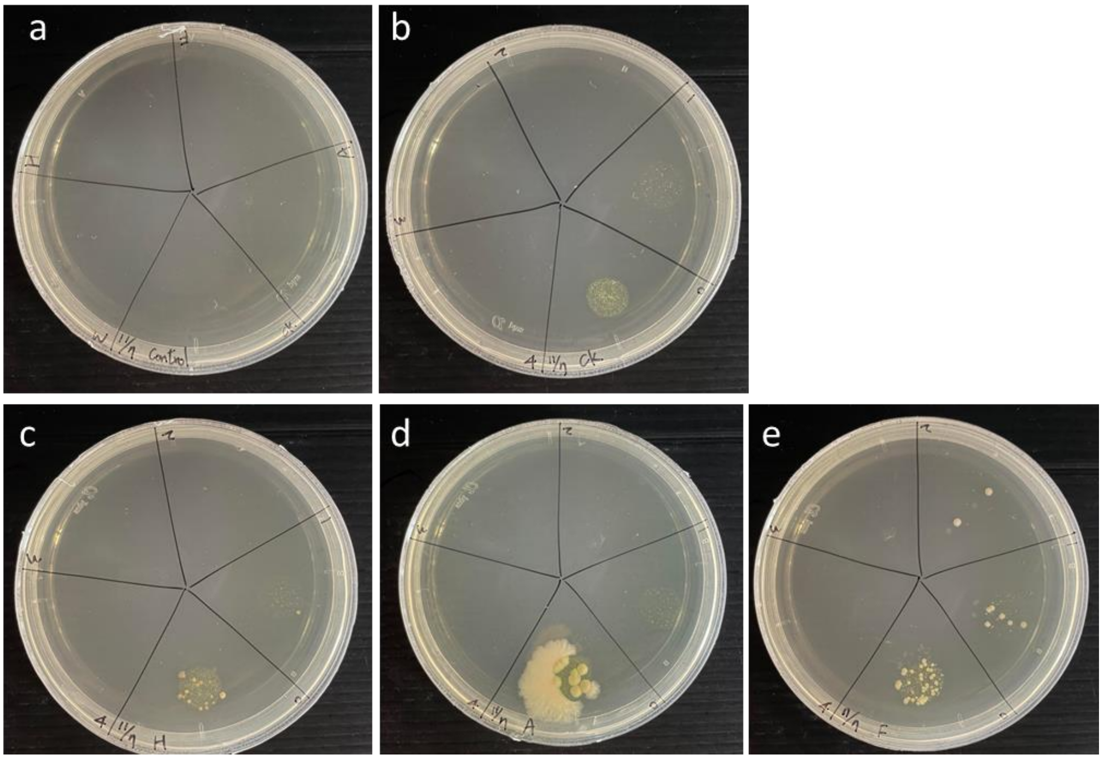

Figure S6. The colonies from supernatant inoculated-*Arabidopsis* plant juices on LB plates.

The LB plates were cultured with sterile distilled water obtained from the final wash of samples (a), *Arabidopsis* plant juices without inoculation (b), HCO3-supernatant inoculation (c), Acetate- supernatant inoculation (d) and FsMSG- supernatant inoculation (e). This image was created by using Microsoft Office Professional 2019 PowerPoint (https://www.microsoft.com/zh-tw/microsoft-365/p/office-%E5%B0%88%E6%A5%AD%E7%89%88-2019/cfq7ttc0k7c5?activetab=pivot%3aoverviewtab).

Table S1. The PQQ-dependent glucose dehydrogenase activity of *E. coli* membrane protein (EcMP) after freeze-thaw cycles.

	Freeze-thaw cycle	PQQ (ppb)	Reaction rate (dAbs/min)
EcMP 50 μg	0	4	-0.0191
	1	4	-0.02184
	3	4	-0.02318
EcMP 100 μg	0	4	0.00863
	1	4	0.00324
	3	4	0.004093
